# Usefulness of texture and color enhancement imaging (TXI) in early gastric cancer found after Helicobacter pylori eradication

**DOI:** 10.1038/s41598-023-32871-3

**Published:** 2023-04-27

**Authors:** Takuya Shijimaya, Tomomitsu Tahara, Tomio Uragami, Narumi Yano, Yutaro Tokutomi, Atsushi Uwamori, Shuhei Nishimon, Sanshiro Kobayashi, Yasushi Matsumoto, Naohiro Nakamura, Takashi Okazaki, Yu Takahashi, Takashi Tomiyama, Yusuke Honzawa, Norimasa Fukata, Toshiro Fukui, Makoto Naganuma

**Affiliations:** Third Department of Internal Medicine, Kansai Medical University, 2-5-1 Shin-Machi, Hirakata, Osaka 573-1010 Japan

**Keywords:** Cancer, Gastroenterology

## Abstract

Early-stage gastric cancer (EGC) found after Helicobacter pylori (Hp) eradication is often difficult to diagnose using conventional white light (WL) endoscopy. We aimed to evaluate whether Texture and Color Enhancement Imaging (TXI), a new image-enhanced endoscopy enhances the EGC lesions after Hp eradication. We also compared diagnostic accuracy and lesion detection time between WL and TXI in trainee endoscopists. 58 EGC lesions after successful Hp eradication were enrolled. Using endoscopic images in WLI, TXI mode 1 (TXI1), and TXI mode 2 (TXI2), visibility of EGC was assessed by six expert endoscopists using a subjective score. Mean color differences (ΔE) of four matched adjacent and intra-tumoral points were examined. Using randomly allocated images, diagnostic accuracy and lesion detection time were evaluated in three trainee endoscopists. Visibility score was unchanged (Score 0) in 20.7% (12/58) and 45.6% (26/57), slightly improved (Score 1) in 60.3% (35/58) and 52.6% (30/57), obviously improved (Score 2) in 45.6% (26/58) and 1.8% (1/57), in TXI1 and TXI2 compared to WL, respectively. Mean ΔE ± SEM in TXI1 (22.90 ± 0.96), and TXI2 (15.32 ± 0.71) were higher than that in WL (1.88 ± 0.26, both *P* < 0.0001). TXI1 presented higher diagnostic accuracy compared to WL, in two of three trainees (94.8% vs. 74.1%, 100% *vs.* 89.7%, *P* = 0.003; < 0.005, respectively). Lesion detection time was shorter in TXI1 in two of three trainees (*P* = 0.006, 0.004, respectively) compared to WL. TXI improves visibility of EGC after Hp eradication that may contribute to correct diagnosis.

## Introduction

Gastric cancer is one of the most common malignancies and the third causal of cancer death worldwide^1^. Although patients who have advanced stage gastric cancer have a poor prognosis, the 5-year disease-specific survival rate for patients with early gastric cancer (EGC) reaches about 90%^2^. Therefore, early detection is important to improve the prognosis of gastric cancer. Endoscopy is a main tool to diagnose gastric cancer and have been widely used in the gastric cancer screening in countries with high incidence of gastric cancer such as Japan and Korea^3,4^. However, detectability of EGC may be affected by the endoscopist’s ability and lesion characteristics, associated with diagnosis difficult, for example, EGC found after Helicobacter pylori (Hp) eradication^5^.

Eradication therapy against Hp infection, which is defined as an obvious carcinogen^6^, has been recommended for the prevention of gastric cancer. However, gastric cancer sometimes occurs even after successful Hp eradication^7,8^, and it is often difficult to detect and diagnose EGC after Hp eradication correctly using conventional white light endoscopy^5^. It has been reported that EGC found after Hp eradication appears as indistinct forms such as tiny and flattened lesions^5,7,8^. Usefulness of image-enhanced endoscopy for the detection of EGC after Hp eradication is recently suggested^9,10^.

Texture and color enhancement imaging (TXI) is a new generation IEE system that is a white light image (WLI)-based imaged enhanced endoscopy^11^. In TXI, an image initially obtained by white-light irradiation is divided into texture and base images. Divided images are recombined after enhancing the texture and correcting the color and brightness. TXI optimizes “structure”, “color”, and “brightness”, of the mucosal surface to better visualize the slight changes in the surface mucosa. Recent studies have reported that TXI enhances the visibility of EGC compared to WL^12,13^. This study aimed to evaluate whether the TXI enhance EGC lesions after Hp eradication. We also compared the diagnostic accuracy and lesion detection time between WL and TXI in trainee endoscopists.

## Materials and methods

### Patients

Prospective cohort from 55 patients with EGC who had history of successful Hp eradication were enrolled in this study. These patients visited to the Endoscopy Center of Kansai Medical University Hospital for the treatment of EGC lesions from June 2021 to August 2022. A history of successful Hp eradication was investigated based on medical records or a face-to-face history examination. Negativity of Hp infection was also confirmed by either of negative urea breath test or negative Hp antigen stool test, and histology of endoscopic biopsy in all patients. Negativity of 2 out of 3 these tests were considered as successful Hp eradication. These 55 patients had 58 EGCs that were diagnosed at least 6 months after eradication (Table 1). The study was conducted in accordance with the ethical principles included in the Declaration of Helsinki and the study protocol was approved from the institutional review board (ID: 2021235). Written informed consents were obtained from all patients.

**Table 1:** Clinicopathological characteristics of patients and gastric neoplasms.

Variables (n)	
Patients: n	55
Lesions: n	58
Gender: male/female	51/7
Age: median years (range)	72 (43–90)
Post-eradication period: median months (range)	78 (6–360)
Reason for Hp eradication: GU/Hp test positive/after ER/unknown	8/37/6/7
Previous history of ESD of gastric neoplasms: yes/no	20/35
Location: Upper third/Middle third/Lower third	6/31/21
Morphology: Depressed/Elevated/Flat	32/21/5
Gastric atrophy: Closed type/Opened type	18/40
Color: Reddish/Whitish/Same as surroundings	33/18/7
Tumor size: mean mm (range)	11.1 (3–40)
Histology: Well/Mod./Por	50/6/2
Invasion depth: T1a/T1b	54/4

*GU* gastric ulcer, *Well* well differentiated adenocarcinoma, *Mod* moderately differentiated adenocarcinoma, *Por.* poorly differentiated adenocarcinoma.

### Endoscopic procedure

Six expert endoscopists (TT, YT, NN, YM, SN, TS) evaluated the gastric neoplastic lesions using the high definition gastroscope (Olympus GIF-H290Z and an EVIS X1 CV-1500; Olympus Medical Systems, Tokyo, Japan). All lesions were evaluated using WL endoscopy, next by TXI mode1 (TXI1), followed by TXI mode 2 (TXI2). Using the WL imaging, morphology and color of lesions were assessed. The degree of atrophic gastritis was evaluated and classified according to the Kimura–Takemoto classification (closed or open type)^14^. Magnifying endoscopy with narrow-band imaging (NBI) was also applied for the detailed assessment of the lesion margin. Although above assessments were based on the each endoscopist who performed independent examination, but its reliability was all confirmed by the consensus manner from two expert endoscopists (TT and TS) reviewing endoscopic images.

The endoscopic system used in the study can promptly change image modalities (WL, TXI 1, TXI2, and NBI) by the push of a button on the scope holder. The structural enhancement function was set to A5 in the WL, TXI1 and TXI2, and to B8 in the NBI.

### Treatment of gastric neoplastic lesions

Endoscopic submucosal dissection (ESD) was carried out in all patients. The resected ESD specimens were fixed in 10% formalin before histopathological assessment. Pathological diagnosis of the resected specimens was carried out according to the Japanese Classification of Gastric Carcinoma, 3rd edition^15^ by expert pathologists in our hospital.

### Endoscopic image data collection

The expert endoscopists (TT and TS) annotated the margin of gastric neoplasms on the WL, TXI1 and TXI2 images by comparing with the magnifying NBI endoscopic image and histological mapping of the ESD specimen. Then, they selected one representative image which included an entire gastric neoplastic lesion taken by WL, TXI1 and TXI2, respectively. Chosen paired image in WL, TXI1, and TXI2 was consistent in distance, angle, and insufflation of the air. Since our goal was to evaluate the feasibility of TXI in lesion detection in screening endoscopy, these images were selected from the middle or distant view. For one lesion, TXI2 image was not available. For this lesion, WLI and TXI1 images were evaluated for the analysis. For remaining 57 lesions, all paired images (WLI, TXI1, and TXI2) were available for analysis.

### Visibility score assessment

Using the representative TXI images, visibility of gastric lesion was evaluated by reference to the WL endoscopy. The visibility of lesion in the TXI image was scored as follows: 0 (visibility equivalent to that of WL), 1 (slightly improved visibility), 2 (obviously improved visibility). In case, the visibility was decreased compared to the WL, we scored them as -1. This assessment was performed by the consensus manner from the six expert endoscopists (TT, YT, NN, YM, SN, TS) comparing the images of lesions by using the WL, TXI1 and TXI2.

### Color difference assessment

Colors of four matched adjacent and intra-tumoral points (proximal, distal, anterior and posterior) were evaluated using the International Commission on Illumination L*a*b*(CIELAB) color space system^16^. The color difference between matched adjacent and intra-tumoral points were evaluated with the formula: ΔE = (ΔL*)2 + (Δa*)2 + (Δb*)21/2. Then, ΔE of four matched adjacent and intra-tumoral points were averaged as the mean ΔE of each lesion.

### Diagnostic yield and lesion detection time in trainee endoscopists

Paired endoscopic images (WL, TXI1, and TXI2) from 58 gastric lesions were randomly allocated. Three trainee endoscopists independently diagnosed these images shown on a laptop monitor in which there was only one real lesion. They were allowed to detect one or more lesions, but real lesion needed to be included in detected lesions. If the real lesion was contained in lesions detected by trainee, we considered it as correct diagnosis. If not, we considered it as wrong diagnosis. The lesion detection time, defined as the time since new image appeared on laptop monitor until endoscopist pointed out last lesion, was also measured using the stopwatch.

### Statistical analysis

Unpaired and paired categorical variables were determined using the Chi-squared test and the McNemar's test, respectively. In case there were two zero cells in 2 × 2 table, the association was evaluated using the binomial test. Unpaired and paired continuous variables were determined using the student's t test and the Wilcoxon signed rank test, respectively. Inter- observer agreement among two different observers was assessed by the Cohen's kappa values of 0, 0.1–0.2, 0.21–0.4, 0.41–0.6, 0.61–0.80, 0.81–0.99, and1 were considered as no, slight, fair, moderate, substantial, near perfect and perfect agreements, respectively. Differences with *P*-values < 0.05 were considered significant.

## Results

### Clinicopathological characteristics patients and gastric neoplasms

Clinicopathological characteristics of 55 patients and 58 gastric neoplasms are shown in the Table 1. The median post-eradication period was 78 months. 20 patients had previous history of ESD for gastric neoplasms such as cancer or adenoma. More than half of the lesions were reddish and depressed morphology, reported as typical appearance of early gastric cancer after Hp eradication^5,7,8^. Histological assessment of resected specimen showed that majority of the lesions were characterized as differentiated type adenocarcinoma.

### Visibility score assessment

**Figure 1:**
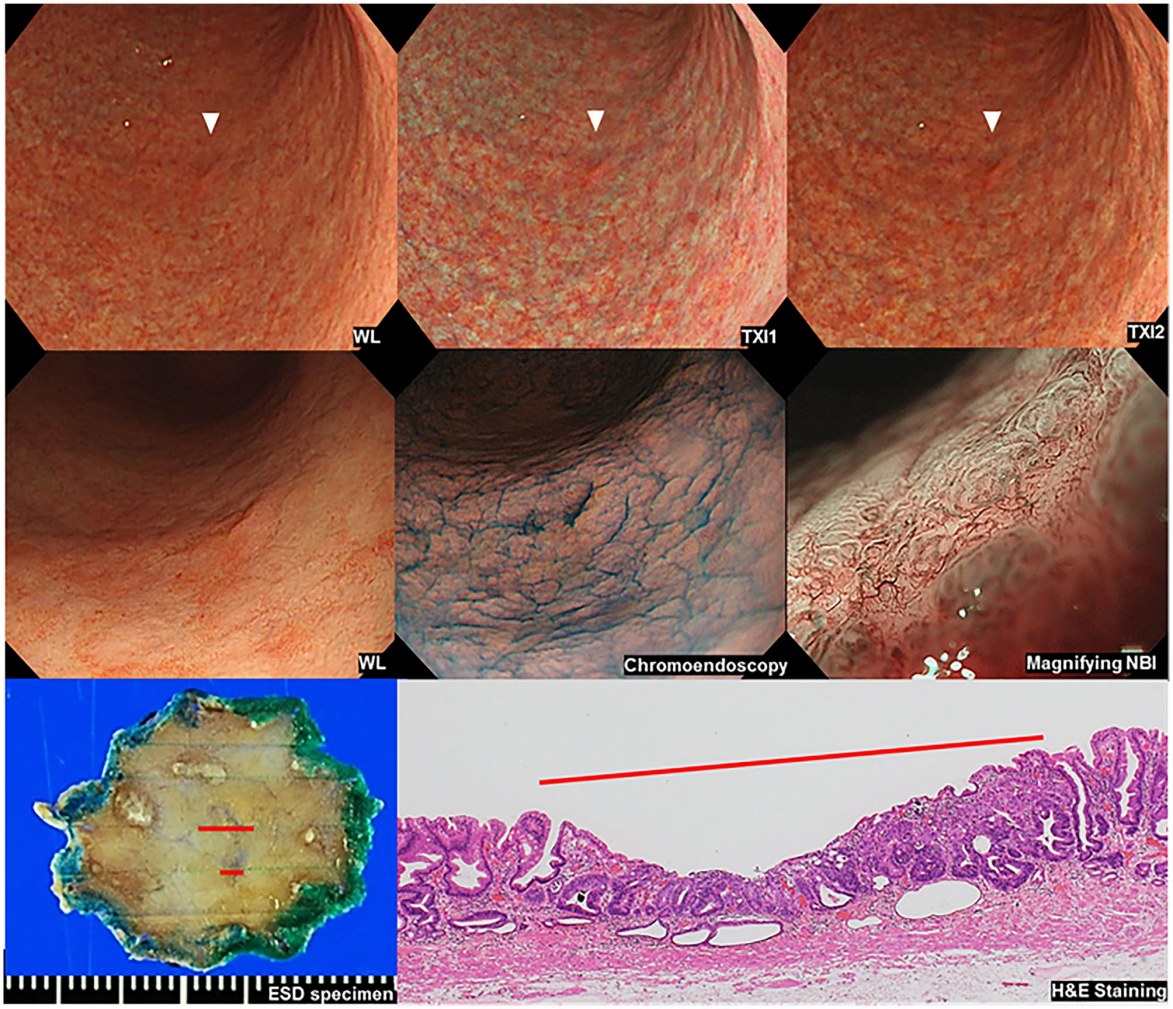
A representative case of EGC with improved visibility by the TXI image. In a distant view, an EGC lesion is observed as slightly reddish color change in the greater curvature of the gastric body but it seems ambiguous in WL image (upper left, white arrow head). TXI mode 1 demonstrated enhancement reddish color (upper middle, white arrow head). TXI mode 2 also demonstrated slight enhancement of reddish color (upper right, white arrow head). In a closer view, the lesion was observed as a depressed lesion (middle left). Chromoendoscopy using indigo carmine enhanced irregular shaped depression (middle center) and magnifying NBI demonstrated irregular micro vessels in the depressed area. Histological assessment of ESD specimen showed well differentiated adenocarcinoma within mucosal layer (red line, lower left and right).

A representative case of EGC with improved visibility by the TXI image was shown in Fig. 1.

The visibility scores rated by the six expert endoscopists are shown in Table 2. Visibility was unchanged (Score 0) in 20.7% (12/58) and 45.6% (26/57), slightly improved (Score 1) in 60.3% (35/58) and 52.6% (30/57), obviously improved (Score 2) in 45.6% (26/58) and 1.8% (1/57), in TXI1 and TXI2 compared to WL respectively. None of the lesions were scored as decreased visibility. The mean visibility scores were 0.98 ± 0.08 and 0.56 ± 0.07 in TXI1 and TXI2, respectively. The mean ± SEM visibility score of TXI1 was significantly higher than that of TXI2 (*P* = 0.0003).

**Table 2:** The visibility scores rated by the six expert endoscopists by the consensus manner.

Variables	Score 0: n (%)	Score 1: n (%)	Score 2: n (%)	Mean score: ± SEM*
TXI 1	12/58 (20.7%)	35/58 (60.3%)	11/58 (19.0%)	0.98 ± 0.08
TXI 2	26/57 (45.6%)	30/57 (52.6%)	1/57 (1.8%)	0.56 ± 0.07

*P = 0.0003, Statistical analysis was performed using the Student's t-test.

TXI2 could not be performed for one lesion.

### Color difference assessment

**Figure 2:**
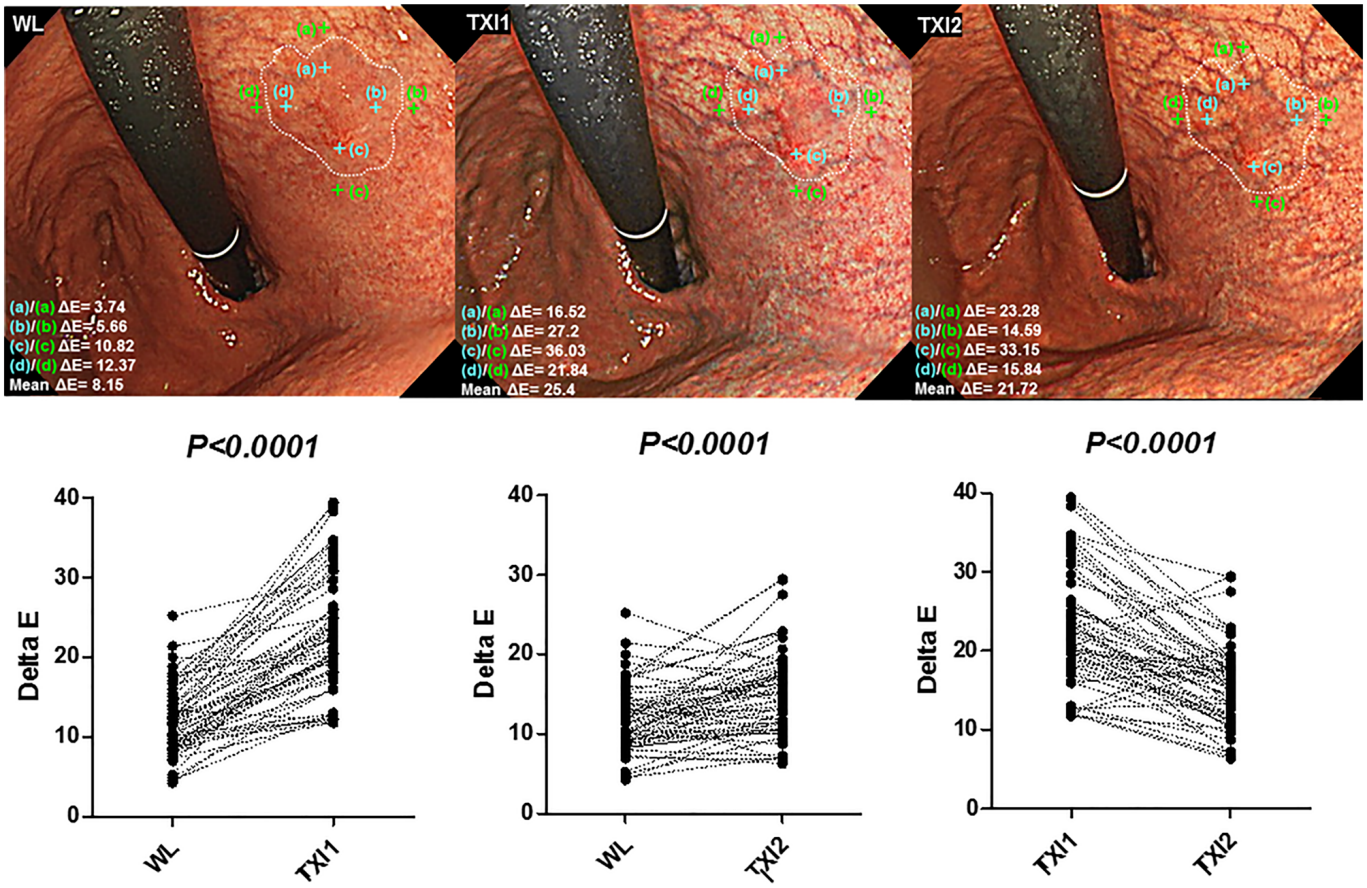
Color difference assessment. Annotation of four matched adjacent and intra-tumoral points and color difference analysis (upper). An EGC lesions is observed as slightly reddish flat area in the lesser curvature of the upper gastric body. Colors of four matched adjacent (light green cross marks) and intra-tumoral points (light blue cross marks) were annotated in proximal, distal, anterior and posterior sites in WL (upper left), TXI1 (upper middle), and TXI2 (upper right) images. Then, ΔE of four matched adjacent and intra-tumoral points were averaged as the mean ΔE of each image. MeanΔE of four matched adjacent and intra-tumoral points were compared between WL, TXI1 and TXI2 (lower). Statistical analysis was performed using the Wilcoxon signed rank test.

The color difference between four matched adjacent and intra-tumoral points (proximal, distal, anterior and posterior) were evaluated. The representative case was shown in Fig. 2.

The mean ΔE ± SEM of the four points in WL, TXI1, and TXI2 was 11.88 ± 0.26, 22.90 ± 0.96, and 15.32 ± 0.71, respectively. The mean ΔE was significantly higher in both TXI1 and TXI2 compared to that of WL (both *P* < 0.0001: Fig. 2).

In the comparison of TXI1 and TXI2, ΔE was significantly higher in TXI1 compared to that of TXI2 (*P* < 0.0001: Fig. 2).

### Diagnostic yield and lesion detection time in trainee endoscopists.

Three trainee endoscopists (trainees A, B and C) independently diagnosed gastric lesions from randomly allocated WL, TXI1, and TXI2 images. All three trainee endoscopists were blinded to patient’s clinical information and image types (WLI or TXI). Detailed diagnostic results and diagnostic accuracies are shown in the Supplementary Table 1 and the Table 3, respectively. The diagnostic accuracy in WL, TXI1, and TXI was 43/58 (74.1%), 55/58 (94.8%) and 51/57 (89.5%) for trainee A, 53/58 (91.4%), 57/58 (98.3%) and 55/57 (96.5) for trainee B and 52/58 (89.7%), 58/58 (100%) and 56/57 (98.2%) for trainee C, respectively (Table 3). The TXI1 presented significantly higher diagnostic accuracy compared to the WL in two of three trainees (trainees A and C, *P* = 0.003, < 0.005, respectively), while the TXI2 presented significantly higher diagnostic accuracy compared to the WL in one of three trainees (trainee A, *P* = 0.04). When the lesion that could be diagnosed in all three trainees was defined as correct diagnosis, overall diagnostic accuracy from 3 trainees was significantly higher in both TXI1 and 2 compared to WL (Both *P* < 0.0001). We also assessed inter- observer agreement assessed by the Cohen's kappa value in WL, TXI1 and 2 across three different trainees. WL presented moderate agreement with kappa values, 0.30–0.50, while TXI1 presented kappa values (0.00–0.03). The TXI2 presented slight to moderate agreement (0.21, 0.66) in two of three trainees but low in one trainee (Supplementary Table 2).

**Table 3:** Diagnostic accuracy of WL, TXI1 and TXI2 across three different trainees.

Variables	Diagnostic accuracy: n (%)	*P*
Trainee A		
WL	43/58 (74.1%)	Reference
TXI1	55/58 (94.8%)	0.003
TXI2	51/57 (89.5%)	0.04
Trainee B		
WL	53/58 (91.4%)	Reference
TXI1	57/58 (98.3%)	0.22
TXI2	55/57 (96.5)	0.37
Trainee C		
WL	52/58 (89.7%)	Reference
TXI1	58/58 (100%)	< 0.005*
TXI2	56/57 (98.2%)	0.13
Over all		
WL	42/58 (72.4%)	Reference
TXI1	54/58 (93.1%)	< 0.0001
TXI2	49/57 (86.0%)	< 0.0001

*Statistical analysis was performed using the binomial test.

All other associations were determined using the McNemar's test.

TXI2 could not be performed for one lesion.

**Figure 3:**
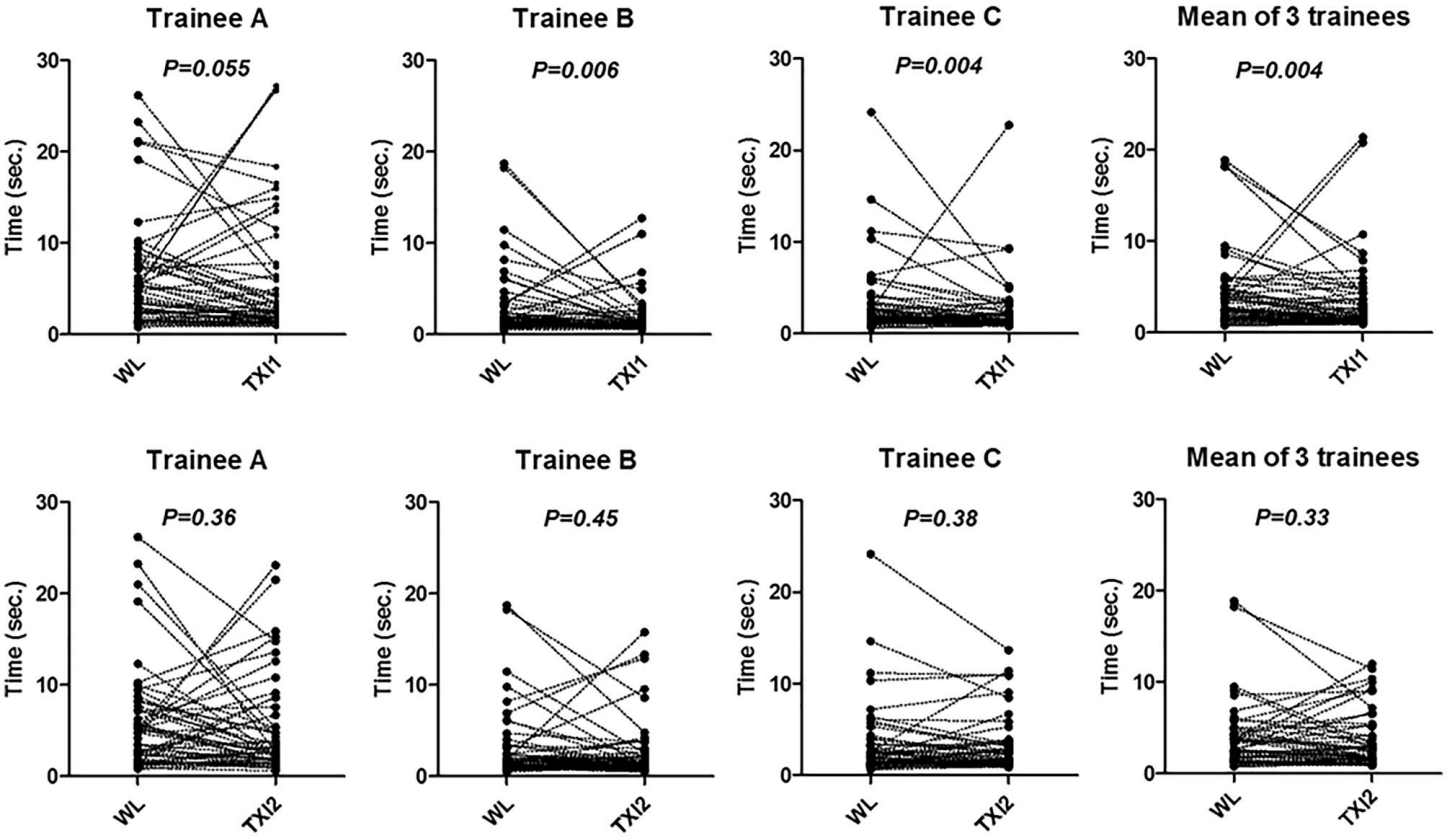
Comparison of lesion detection time between WL, TXI1 and TXI2 in three trainee endoscopists. Statistical analysis was performed using the Wilcoxon signed rank test.

We next compared the lesion detection time, defined as the time since new image appeared on laptop monitor until endoscopist pointed out all lesions. We found that the lesion detection times of two of three trainees as well as its mean of three trainees were significantly shorter in TXI1 (trainees B, C and mean of three trainees, *P* = 0.006, 0.004, 0.004, respectively), compared to that in WL (Fig. 3). For one trainee (trainee A), the lesion detection time was also tended to be shorter in TXI1 compared to that in WL (trainee A, *P* = 0.055). On the other hand, there was no difference in lesion detection time in TXI2 compared to that in WL (Fig. 3).

### Association between usefulness of TXI1 endoscopy and clinicopathological characteristics of gastric tumor

We then investigated whether any clinicopathological characteristics of gastric neoplasms would be associated with usefulness of TXI1 image. The lesion which could not be diagnosed at least one or more trainee endoscopists in WL but could be diagnosed in all three trainee endoscopists in TXI1 images were identified as TXI1 lesion. Then, association between TXI1 lesion and clinicopathological characteristics of gastric tumor was investigated (Table 4). We found that TXI1 lesion was significantly associated with color of tumor, such as same color as surroundings (*P* = 0.03). Weak assocation was also found between TXI1 lesion and morphology of tumors such as flat type (*P* = 0.05). Remaining clinicopathological characteristics, such as location, degree of gastric atrophy, histology, invasion depth, post-eradication period and lesion size, were not significantly associated with TXI1 lesion.

**Table 4:** Association between usefulness of TXI1 endoscopy and clinicopathological characteristics of gastric tumor.

Variables (n)	TXI1 lesion (14)	Other (44)
Location		
Upper third (6)	0	6
Middle third (31)	8	23
Lower third (21)	6	5
Morphology*		
Depressed (32)	9	23
Elevated (21)	2	19
Flat (5)	3	2
Degree of gastric atrophy		
Closed type (18)	2	16
Opened type (40)	12	28
Color**		
Reddish (33)	7	26
Whitish (18)	3	15
Same color as surroundings (7)	4	3
Histology		
Well differentiated adenoca. (50)	13	37
Moderately differentiated adenoca. (6)	1	5
Poorly differentiated adenoca. (2)	0	2
Invasion depth		
T1a (54)	14	40
T1b (4)	0	4
Post-eradication period: mean ± SEM	80.8 ± 18.3 months	80.8 ± 18.3 months
Tumor size: mean ± SEM	12.6 ± 3.3 mm	10.6 ± 0.9 mm

TXI1 lesions, lesions at least one trainee could not detect lesion by the WL but all trainees could detect lesion by using the TXI1; adenoca., adenocarcinoma.

*Flat vs. others, *P* = 0.05; **same as surroundings vs. others, *P* = 0.03.

Categorical and continuous variables were determined using the Chi-squared test and the student's t test, respectively. Post-eradication period was not determined for five cases.

## Discussion

We evaluated the diagnostic feasibility of TXI imaging in the diagnosis of ECG after Hp eradication. The visibility was evaluated by the subjective score and theΔE color difference value. In our study, TXI1 showed the highest visibility score compared to that of WL among six expert endoscopists. This result was highlighted by the highest ΔE color difference value in TXI1 compared to that is WL and TXI2. Our data suggest that TXI1 provides better brightness and enhancement of texture and color to emphasize slight changes in the surface mucosa seen in EGC after Hp eradication. In principal, TXI2 enhances texture and brightness, and TXI mode1 further enhances color. Therefore, the color contrast between red and white is greater under TXI1 than in TXI2. There were two former studies that reported visibility of EGC in TXI^11,12^. Both of them reported highest ΔE color difference value in TXI1 compared to that in the WL, and TXI2, which was in line with our result. Concerning the TXI2, Ishikawa et al. investigated the color difference between non-neoplastic and neoplastic areas of 12 gastric neoplasms (carcinoma and adenoma) and showed that ΔE value in the TXI1 was higher than that in TXI2^11^. Abe et al. also investigated the same issue in 20EGC lesions and reported that there was no significant difference in ΔE values between TXI1 and TXI2^12^. In our data, ΔE value in the TXI2 was lower than that in TXI1 but still higher than that in WL. This discrepancy may be mainly due to the differences in sample size and statistical method. The sample sizes seen in other studies were relatively small. In addition, our study and Ishikawa’s study processed result by the paired statistical test (Wilcoxon signed rank test), while Abe’s study processed result by the unpaired statistical test, which may influence the* P* value. Our data suggest that both TXI1 and TXI2 improve visibility of EGC lesions, but TXI1 is superior to TXI2.

To further evaluate the potential usefulness of TXI in the diagnosis of EGC after Hp eradication, we compared the diagnostic accuracy and lesion detection time between WL and TXI in trainee endoscopists. The TXI1 presented significantly higher diagnostic accuracy and shorter lesion detection time in two of three trainees. These results suggested the usefulness of TXI1 image in the diagnosis of gastric neoplasms after Hp eradication, which is applicable for trainee endoscopists. We showed that EGC lesion, which the TXI1 was better than WL, was significantly associated with EGC with the same color as surrounding mucosa. Similar result was also found in the flat type lesion. EGC found after Hp eradication appears as indistinct forms such as tiny and flattened lesions, and it is often difficult to detect and diagnose correctly using conventional WL^5,7,8^. The result suggests that TXI1 provides better brightness, enhancement of texture and color in ambiguous EGC lesions after Hp eradication. Result from trainees also supports the evidence that TXI is effective for EGC detection regardless of endoscopist’s ability. When considering the inter- observer agreement in different trainees, however, the agreement was rather low in TXI1 compared to WL and TXI2, which was unexpected but may be due to the high diagnostic accuracy of TXI1 especially in two trainees (trainee B and C, 98.3% and 100%, respectively).

Concerning other image-enhanced endoscopies in the diagnosis of EGC, blue light imaging (BLI) made from the combination of a 410 nm wave length strong laser light and a 450 nm wavelength weak laser light, was reported to have a higher real-time EGC detection rate than WL especially in Hp negative EGC after eradication^10^. Linked color imaging (LCI) is a similar image enhanced technology to TXI that provides enhanced color differences in mucosal color, allowing better lesion recognition with sufficient brightness. A cross-sectional study reported that visibility score of EGC from expert endoscopists was significantly higher in LCI than in WLI, while the study did not evaluate about EGC detection^9^. Another randomized controlled trial demonstrated that LCI is more effective than WLI for detecting neoplastic lesions in the pharynx, esophagus, and stomach, however, in subgroup, the result did not achieve significant difference in the gastric neoplastic lesion detection rates probably due to insufficient sample size^17^. Other randomized controlled trial evaluated the EGC detection rate between WL and new-generation NBI, but new-generation NBI did not improve EGC detection compared to WL^18^. Therefore, utility of image-enhanced endoscopy for detection of EGC in the screening endoscopy remains unclear.

Our result suggests that TXI1 better enhances EGC lesions and effective for EGC detection after Hp eradication especially in trainee endoscopists. This is the first evidence that TXI is useful in the diagnosis of EGC after Hp eradication. However, this study was image-based analysis that was not performed in real clinical setting. Assessment of visibility score and color difference as performed by retrospective manner, which may lead to subjective bias. We used randomly allocated images for evaluation of EGC detection in trainee endoscopists but selection bias, such as carryover effects could not be completely avoided. At this preliminary stage, our study mainly focused on the evaluation of visibility of EGC lesions after Hp eradication, which did not include non-cancerous lesions. We could not evaluate sensitivity, specificity, positive and negative predictive values. Furthermore, to assess the inter-observer agreement more accurately, further case accumulation including diagnostic difficult lesions using the TXI1 will be needed. TXI1 might be helpful in diagnosis of EGC after Hp eradication but it demands further investigation. Large well-designed clinical trials are therefore warranted to investigate the performance of TXI during real-time EGC screening.

## Supplementary Information

Supplementary Tables.

## Data Availability

All data generated or analyzed during this study are included in this published article [and its supplementary information files].

## References

[1] Ferlay J (2019). Estimating the global cancer incidence and mortality in 2018: GLOBOCAN sources and methods. Int. J. Cancer.

[2] Kakeji, Y. *et al.* Registration Committee of the Japanese Gastric Cancer Association. A retrospective 5-year survival analysis of surgically resected gastric cancer cases from the Japanese Gastric Cancer Association nationwide registry (2001–2013). *Gastric Cancer* **25**, 1082–1093 (2022). 10.1007/s10120-022-01317-6 35790645

[3] Hamashima C (2018). Update version of the Japanese guidelines for gastric cancer screening. Jpn. J. Clin. Oncol..

[4] Jun JK (2017). Effectiveness of the Korean National Cancer Screening Program in reducing gastric cancer mortality. Gastroenterology.

[5] Saka A (2016). Endoscopic and histological features of gastric cancers after successful Helicobacter pylori eradication therapy. Gastric Cancer.

[6] Herrero R (2014). The fight against gastric cancer—The IARC Working Group report. Best Pract. Res. Clin. Gastroenterol..

[7] Kamada T (2005). Effect of long-term half-dose famotidine therapy on corpus gastritis in peptic ulcer disease. Aliment. Pharmacol. Ther..

[8] Yamamoto K (2011). Clinicopathological analysis of early-stage gastric cancers detected after successful eradication of Helicobacter pylori. Helicobacter.

[9] Matsumura S (2021). Improved visibility of early gastric cancer after successful helicobacter pylori eradication with image-enhanced endoscopy: A multi-institutional study using video clips. J. Clin. Med..

[10] Dohi O (2019). Blue laser imaging-bright improves the real-time detection rate of early gastric cancer: A randomized controlled study. Gastrointest. Endosc..

[11] Sato T (2021). TXI: Texture and color enhancement imaging for endoscopic image enhancement. J. Healthc. Eng..

[12] Ishikawa T (2021). Efficacy of Texture and Color Enhancement Imaging in visualizing gastric mucosal atrophy and gastric neoplasms. Sci. Rep..

[13] Abe S (2021). Visibility of early gastric cancer in texture and color enhancement imaging. DEN. Open.

[14] Kimura K, Takemoto T (1969). An endoscopic recognition of atrophic border and its signifcance in chronic gastritis. Endoscopy.

[15] Japanese Gastric Cancer Association (2011). Japanese classification of gastric carcinoma: 3rd English edition. Gastric Cancer.

[16] Kuehni RG (1976). Color-tolerance data and the tentative CIE 1976 L a b formula. J. Opt. Soc. Am..

[17] Ono S (2021). Linked color imaging focused on neoplasm detection in the upper gastrointestinal tract: A randomized trial. Ann. Intern. Med..

[18] Yoshida N (2021). Early gastric cancer detection in high-risk patients: A multicentre randomised controlled trial on the effect of second-generation narrow band imaging. Gut.

