# Supplementary material for: Usefulness of texture and color enhancement imaging (TXI) in early gastric cancer found after Helicobacter pylori eradication
Source: Sci Rep. 2023 Apr 27;13:6899. doi: 10.1038/s41598-023-32871-3 (PMC10140262; doi:10.1038/s41598-023-32871-3)
Supplement: Supplementary file 1 — Supplementary Tables. [file 41598_2023_32871_MOESM1_ESM.docx]

**Supporting information**

| **Table S1.** Detailed results from three trainees | | |
| --- | --- | --- |
| in the diagnosis of gastric lesions by WL, | | |
| TXI1 and TXI2 images | |  |
| *Trainee A* |  |  |
|  | TXI1 correct | TXI1 wrong |
| WL correct | 42 (72.4%) | 1 (1.7%) |
| WL wrong | 13 (22.4%) | 2 (3.4%) |
|  |  |  |
|  | TXI2 correct | TXI2 wrong |
| WL correct | 39 (68.4%) | 3 (5.3%) |
| WL wrong | 12 (21.1%) | 3 (5.3%) |
|  |  |  |
| *Trainee B* |  |  |
|  | TXI1 correct | TXI1 wrong |
| WL correct | 52 (89.7%) | 1 (1.7%) |
| WL wrong | 5 (8.6%) | 0 (0%) |
|  |  |  |
|  | TXI2 correct | TXI2 wrong |
| WL correct | 51 (89.5%) | 1 (1.8%) |
| WL wrong | 4 (7.0%) | 1 (1.8%) |
|  |  |  |
| *Trainee C* |  |  |
|  | TXI1 correct | TXI1 wrong |
| WL correct | 52 (89.7%) | 0 (0%) |
| WL wrong | 6 (10.3%) | 0 (0%) |
|  |  |  |
|  | TXI2 correct | TXI2 wrong |
| WL correct | 50 (87.7%) | 1 (1.8%) |
| WL wrong | 6 (10.5%) | 0 (0%) |
| TXI2 could not be performed for one lesion. | | |

| **Table S2** Inter- observer agreement assessed by the Cohen's kappa | | | |
| --- | --- | --- | --- |
| value in WL, TXI1 and 2 across three different trainees | | |  |
| Variables | WL | TXI1 | TXI2 |
| Trainee A/Trainee B | 0.31 | 0.03 | 0.21 |
| Trainee A/Trainee C | 0.50 | 0.00 | 0.03 |
| Trainee B/Trainee C | 0.30 | 0.00 | 0.66 |
